# Systems-based psychiatry: insights from psychedelic research on mechanisms of healing

**DOI:** 10.3389/fpsyt.2026.1789902

**Published:** 2026-07-13

**Authors:** Scott Shannon, Andrew Weil

**Affiliations:** 1 Wholeness Center, Fort Collins, CO, United States; 2 Andrew Weil Center for Integrative Medicine, University of Arizona, Tucson, AZ, United States

**Keywords:** complexity science, integrative medicine, network neuroscience, psychedelic medicine, psychedelic-assisted therapy, psychological flexibility, psychopharmacology, systems-based psychiatry

## Abstract

Current psychiatric models, while clinically valuable, remain limited in their ability to account for the complexity, variability, and context dependence of mental disorders. Converging evidence from neuroscience, complexity science, and psychedelic research suggests that psychiatric symptoms may be better understood as stable patterns of organization within dynamic, multilevel systems rather than as outputs of isolated biological dysfunction. This paper proposes a systems-based framework in which therapeutic change is conceptualized as a phased process of perturbation, reorganization, and consolidation. Within this model, interventions act by modulating system stability and plasticity, with outcomes determined by interactions among biological, psychological, relational, and environmental factors over time. Psychedelic-assisted therapies provide a particularly revealing case, as they reliably induce transient destabilization of entrenched patterns, increasing flexibility and enabling reorganization under supportive conditions. This formulation suggests a generalizable mechanism of healing across modalities: the strategic destabilization of rigid system states followed by the consolidation of more adaptive configurations. Accordingly, recovery is reframed not as symptom reduction alone, but as increased coherence, flexibility, and adaptive capacity across domains of functioning. This perspective has implications for assessment, ethics, clinical implementation, and research design, including the need for longitudinal, context-sensitive outcome measures and hybrid methodologies. A systems-based psychiatry does not replace mechanistic models but reorganizes them within a framework that accommodates emergence, temporal dynamics, and context, offering a foundation for more integrative and preventive approaches to mental health care.

## Highlights

- Psychiatric healing often reflects multilevel reorganization (brain, mind, relationships, and context), not just correction of isolated molecular problems.
- Psychedelic research challenges assumptions about chronic dosing, mechanisms of action, and how acute drug effects relate to long-term benefit.
- Ketamine and psychedelics show that brief interventions can produce lasting change, especially when paired with appropriate therapeutic support.
- A systems-based approach focuses on identifying what maintains rigidity, then sequencing multimodal treatments to support adaptive change rather than symptom suppression alone.
- Safe, scalable implementation will require workforce training, practical assessment tools, and pragmatic trials that test medication–therapy interactions.

## 1 Introduction: psychiatry at a moment of conceptual instability

Philosophy structures science, and science drives medicine. Together they shape our understanding of the psyche and, in turn, our approach to mental health care (1). Yet most clinicians rarely examine the philosophical assumptions underlying psychiatric practice, not out of neglect, but because training often treats these assumptions as settled fact rather than as the conceptual scaffolding upon which evidence, diagnosis, and treatment rest (2) (3) (4). What we call mechanism, evidence, or disorder always reflects a prior conceptual frame (5, 6). As a result, explanatory models often persist even as their clinical adequacy erodes (7, 8).

These assumptions are increasingly strained in everyday clinical practice. Psychiatrists frequently encounter treatment resistance, partial response, relapse, and chronic symptom persistence despite adherence to evidence-based pharmacologic approaches (9–11). Conditions such as major depressive disorder, posttraumatic stress disorder, and anxiety disorders often follow recurrent courses, with diminishing returns across successive interventions (12–14). Improvements in symptoms do not consistently translate into durable gains in functioning, relational capacity, or quality of life (15–17). This gap between symptom reduction and sustained recovery remains a central challenge in contemporary psychiatric care.

It is against this backdrop that psychiatry can be understood as entering a period of conceptual transition. Models centered on isolated biological mechanisms have proven insufficient to account for the heterogeneity and context sensitivity of many psychiatric conditions, prompting renewed attention to developmental, relational, and systemic dimensions of illness and recovery (18, 19). Importantly, this shift does not reject biology; rather, it situates biological processes within dynamic, multilevel systems whose behavior emerges from interactions across neural, psychological, and environmental domains over time (20, 21).

Modern scientific medicine took shape when inquiry was restricted to what could be reliably measured. While this approach yielded major advances, it narrowed the scope of psychiatry by privileging observable mechanisms over subjective experience, meaning, and context (22–24). The psyche came to be modeled as a biological system to be corrected, and treatment as the repair of malfunctioning parts. This framework continues to shape research design and clinical care.

At the same time, clinical observation suggests that psychiatric change often involves coordinated shifts across neural, psychological, relational, and contextual domains (25). Trauma, relational experience, and meaning-making processes all influence outcomes in ways that are not easily explained by linear or single-level models (26). These patterns point toward a view of the mind as a dynamic, self-organizing system rather than a collection of isolated components (20).

Across multiple treatment modalities including psychotherapy, ketamine, and psychedelic-assisted interventions, clinical change can often follow a sequence of perturbation, reorganization, and consolidation (27, 28). Perturbation refers to a temporary destabilization of established patterns; reorganization to the emergence of new configurations; and consolidation to the stabilization of these changes over time through behavioral, relational, and contextual reinforcement (28).

Engel’s biopsychosocial model provided an important foundation for integrating multiple domains of influence. However, it has often functioned descriptively rather than as a dynamic account of how change unfolds (29, 30). A systems-based approach extends this framework by emphasizing nonlinear interactions, temporal sequencing, and the role of system-level constraints in shaping both pathology and recovery (31).

This article advances the hypothesis that psychiatric interventions can be understood as perturbational inputs into complex, self-organizing systems, and that durable outcomes depend less on sustained molecular effects than on the timing, context, and integration of these perturbations within multilevel systems of regulation. From this perspective, psychiatric illness can be understood to reflect patterns of constrained organization, and treatment involves the strategic modulation of system stability and plasticity over time.

While several theoretical frameworks have addressed components of this perspective, including the REBUS model of psychedelic brain action, network theories of psychopathology, and predictive processing accounts of psychiatric symptoms, none has systematically linked these neuroscientific models to a phased clinical framework specifying how stabilization, perturbation, and consolidation should be sequenced and coordinated across treatment domains (20, 32). This paper attempts to bridge that gap by proposing a clinically oriented systems model that maps the perturbation-reorganization-consolidation sequence onto concrete phases of psychiatric care (1): system assessment, including evaluation of stability, constraint patterns, and adaptive capacity (2); preparatory stabilization, in which biological, relational, and environmental conditions are optimized to support safe perturbation (3); strategic perturbation, timed to coincide with sufficient system coherence; and (4) integration and consolidation, in which emerging patterns are reinforced through psychotherapy, behavioral change, and contextual support. This phased model is not specific to psychedelic-assisted therapy but applies across interventions that modulate system plasticity, including ketamine (33), neuromodulation (34), and intensive psychotherapeutic approaches (28). Its contribution is therefore not a new neuroscientific theory but a translational framework that connects existing theoretical advances to the practical structure of psychiatric care.

Several existing frameworks address components of the perspective advanced here and clarifying the relationship between them is essential to specifying the contribution of a systems-based clinical model. The three-phase trauma treatment model: stabilization, trauma processing, and reintegration that has been articulated by Herman and widely adopted in PTSD care, represents an important clinical precedent that shares structural similarities with the stabilization-perturbation-consolidation sequence proposed in this paper (35). However, the trauma model was developed primarily for a single diagnostic context and does not provide a transdiagnostic account of how system-level dynamics govern the timing, safety, and durability of change across intervention modalities.

Similarly, the REBUS model offers a compelling neuroscientific account of how psychedelics alter predictive processing hierarchies, but it is principally a model of acute psychedelic brain action rather than a clinical framework specifying how perturbation should be prepared for, sequenced, and consolidated across biological, psychological, and relational domains over time (27). Network theories of psychopathology, as developed by Borsboom and colleagues, conceptualize symptoms as mutually reinforcing elements within dynamic systems and have generated productive empirical research programs; yet these models have focused primarily on symptom-level network structure rather than on the temporal phasing of clinical interventions or the role of contextual and relational factors in shaping system reorganization (32).

The framework proposed here does not supersede these contributions but attempts to integrate their insights into a unified, clinically oriented model that specifies how stabilization, perturbation, and consolidation should be coordinated across treatment domains and across diagnostic boundaries, a translational layer that, to date, has not been systematically articulated in the literature. Finally, the inclusion of ketamine and electroconvulsive therapy as perturbational analogues warrants qualification: ketamine’s antidepressant effects typically require repeated administration to sustain benefit, whereas psilocybin has demonstrated durable improvements following one or two sessions, a divergence that may reflect differences in the depth or nature of system-level reorganization elicited by each agent and that merits further empirical investigation rather than premature unification under a single mechanistic umbrella.

This framework has implications for both research and practice. It suggests that assessment should extend beyond diagnosis to include mapping of system-level constraints; that treatment should be conceptualized as a sequence of stabilizing and destabilizing interventions; and that outcomes should be evaluated over extended time horizons, incorporating functional, relational, and meaning-based measures in addition to symptom change (36–39). A number of testable interventions can be derived from this model. A few are listed below.

### 1.1 Testable Hypotheses Derived From This Framework

1. Sequencing hypothesis: In patients with treatment-resistant depression and high baseline instability (low Heart Rate Variability, high network rigidity), stabilizing interventions (sleep optimization, relational support) delivered *before* ketamine or psychedelic therapy will produce more durable symptom reduction than destabilizing interventions introduced without preparation (testable via stepped-wedge Randomized Controlled Trial).
2. Plasticity window hypothesis: Psychotherapeutic interventions (e.g., cognitive restructuring, values clarification) delivered during the subacute post-psychedelic period (days 3-14) will produce larger effect sizes than identical interventions delivered before or after this window (testable via dismantling design).
3. System flexibility hypothesis: Durable clinical response will correlate more strongly with post-treatment changes in psychological flexibility and network flexibility (fMRI entropy) than with acute symptom reduction at week 1 (testable via mediation analysis in existing datasets).
4. Constraint reduction hypothesis: In resource-limited settings, targeted reduction of maintaining constraints (e.g., sleep, nutrition, social isolation) via low-cost behavioral interventions will produce non-inferior outcomes compared to added pharmacotherapy in mild-moderate depression (testable via pragmatic equivalence trial).

## 2 The mechanistic paradigm in psychiatry: achievements and emerging limits

Psychiatry’s development over the past century reflects both the strengths and constraints of mechanistic scientific thinking. Reductionist models yielded major advances, particularly in severe mental illness, by identifying biological targets and developing pharmacologic interventions that reduced suffering and stigma (1). Framing psychiatric disorders as medical conditions represented an important ethical and clinical advance.

At the same time, the explanatory sufficiency of narrowly mechanistic models has been increasingly questioned. Contemporary reviews indicate that simple neurotransmitter-deficiency frameworks cannot adequately explain the heterogeneity, chronicity, or variable treatment response observed in disorders such as major depression (40, 41). Parallel advances in trauma science, developmental neuroscience, and network neuroscience suggest that psychiatric symptoms often reflect maladaptive organization across interacting neural, physiological, psychological, and contextual systems rather than isolated molecular abnormalities (19, 21). Many conditions cannot be fully explained or effectively addressed by reductionistic models emphasizing isolated molecular targets alone, prompting growing interest in systems-based psychiatry, complexity science, and network neuroscience that situate biological mechanisms in dynamic multilevel frameworks (42).

Although psychiatry articulated a systems-oriented vision with Engel’s biopsychosocial model in the 1970s, prevailing care structures have continued to favor brief, medication-centered encounters (23, 43). This pattern is often accentuated by reimbursement structures as well. As a result, contemporary practice often diverges from contemporary science, prioritizing symptom suppression over broader system assessment and reorganization (44–46). This gap provides essential context for the relevance of emerging research paradigms.

Psychedelic research does not resolve these structural tensions, nor does it replace existing treatments. Rather, it offers a particularly clear illustration of how psychiatric change may emerge through coordinated reorganization across biological, psychological, relational, and contextual domains—rendering visible processes often obscured within mechanistic frameworks (47–49).

## 3 Complexity science and the emergence of systems-based models

Across biology, neuroscience, and medicine, there has been a gradual shift from strictly mechanistic models toward frameworks grounded in complexity science. Rather than viewing organisms as collections of independently functioning parts, complexity science conceptualizes living systems as dynamic, self-organizing entities whose behavior emerges from interactions across multiple levels (20, 50, 51). These systems operate far from thermodynamic equilibrium, maintaining organized structure through continuous exchange with their environment rather than through static stability (50, 52). From this perspective, health and pathology reflect patterns of organization, flexibility, and adaptive capacity rather than isolated defects.

Importantly, a systems-based approach does not replace mechanistic explanations but situates them within a broader context. Molecular, cellular, and circuit-level processes remain essential; however, their effects depend on how they interact within larger networks over time. This distinction helps reconcile two observations in psychiatry: that biological mechanisms clearly contribute to mental illness, and that no single mechanism adequately explains its variability, chronicity, or responsiveness to treatment (18). This framework does not replace mechanistic explanations but provides a structure for integrating them into clinically actionable, multilevel formulations (51).

In neuroscience, this shift is reflected in network-level models demonstrating that psychiatric symptoms are more consistently associated with patterns of activity across large-scale functional systems than with discrete neurochemical abnormalities (32, 53). Conditions such as major depression and posttraumatic stress disorder can be conceptualized as relatively stable patterns of network organization, often described as attractor states, that constrain thought, emotion, and behavior (20). These states are not fixed lesions but dynamic configurations that persist because they are self-reinforcing.

**Figure 1:**
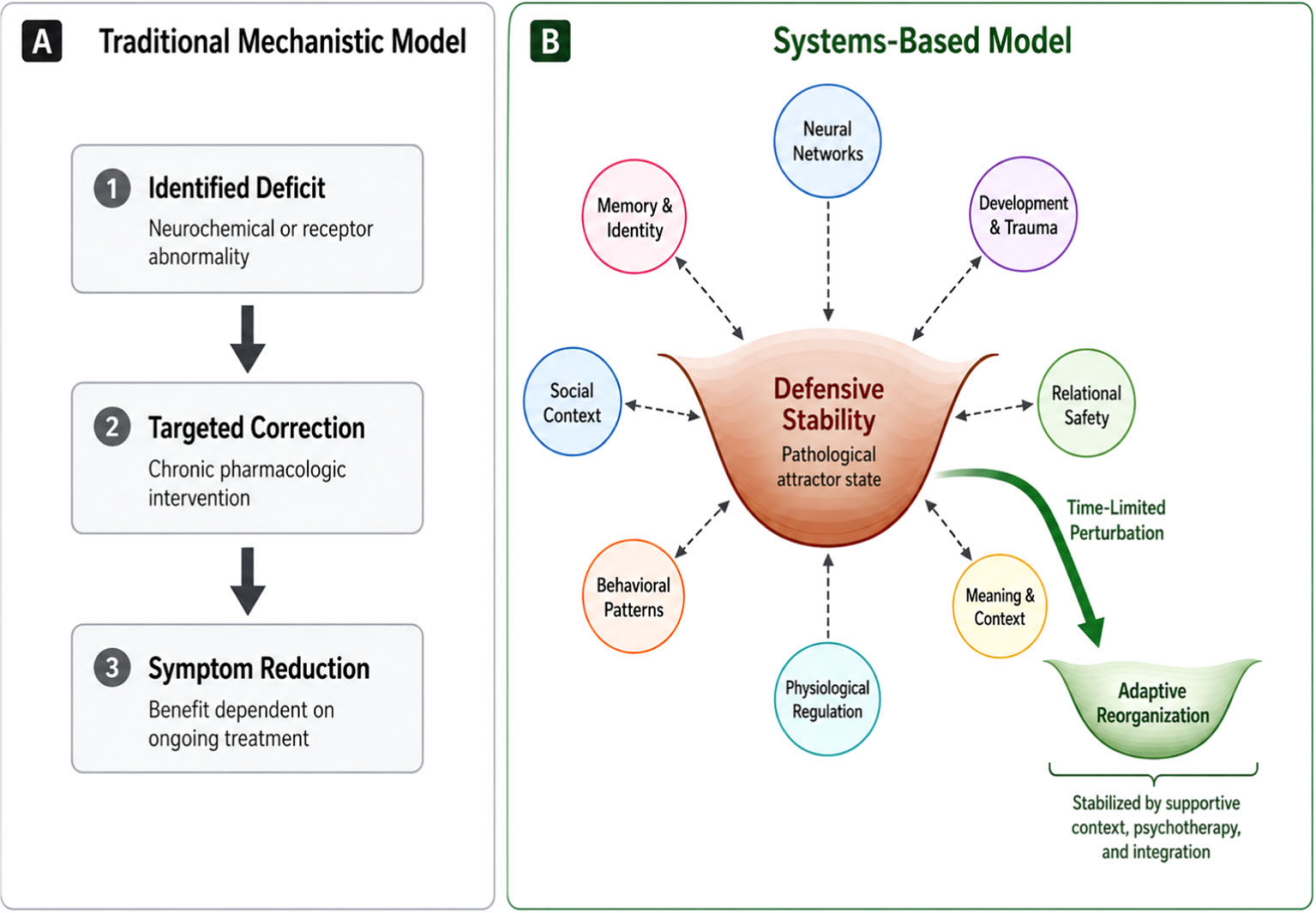
Conceptual comparison of mechanistic and systems-based models of psychiatric healing. **(A)** Traditional mechanistic model. Psychiatric symptoms are conceptualized as the downstream expression of an identified biological defect, such as a neurochemical or receptor abnormality. Treatment is framed as targeted correction through ongoing pharmacologic intervention, with symptom reduction dependent on continued treatment exposure. **(B)** Systems-based model. Psychiatric symptoms are conceptualized as a pathological attractor state, represented by a deep basin of defensive stability maintained by mutually reinforcing interactions across neural, developmental, relational, physiological, behavioral, social, autobiographical, and meaning-making domains. A time-limited perturbation may temporarily destabilize this rigid configuration, allowing the system to reorganize into a more adaptive, shallower attractor state characterized by greater flexibility and lower maintenance costs. Supportive context, psychotherapy, and integration help stabilize the new pattern and reduce the likelihood of returning to the prior attractor state.

Developmental research further supports this view. Early adversity influences neural connectivity, stress physiology, immune signaling, and cognitive-emotional processing across the lifespan (54–56). These changes do not localize to a single system but are distributed across interacting domains, shaping how individuals respond to internal and external demands over time (57). Psychopathology, in this context, reflects patterns that are maintained through feedback loops linking biology, experience, and environment rather than through a single causal pathway (58). See **Figure 1** for a simplisitc visual example of this change from linear mechanism to systems based mechanism. Developmental neuroscience provides a parallel illustration of these dynamics, as infant cognitive and emotional development frequently proceeds through transient periods of behavioral destabilization, including sleep disruption, emotional dysregulation, and regression-like phenomena, that often precede the emergence of new adaptive capacities, supporting the broader principle that nonlinear instability may be intrinsic to developmental reorganization rather than merely a sign of dysfunction (26, 36, 59).

Within this framework, psychiatric change can be understood as a shift in system organization. Rigid or maladaptive patterns may persist because the system lacks sufficient flexibility to transition into alternative configurations. Interventions may therefore act not only by modifying specific biological targets, but by altering the stability of these patterns. This provides a theoretical basis for perturbation-reorganization processes, in which a temporary disruption of established dynamics allows the system to reorganize into a new state (20, 28). The durability of change then depends on whether new patterns are reinforced across biological, psychological, and relational domains (60).

This perspective extends, but differs from, the biopsychosocial model. While the biopsychosocial approach identifies multiple relevant domains, it does not specify how these domains interact over time to produce stable or changing patterns of functioning (51, 61). A systems-based model emphasizes these interactions explicitly, focusing on feedback processes, temporal sequencing, and the conditions under which systems become rigid, unstable, or adaptive.

Crucially, the biopsychosocial model has functioned primarily as a descriptive framework acknowledging multiple contributing factors, but it has not generated operationalizable predictions about when and how to sequence interventions, nor has it specified measurable indices of system rigidity, plasticity, or constraint. In practice, contemporary psychiatry has largely implemented the biopsychosocial model as parallel assessments (diagnosis + psychosocial factors) rather than as a dynamic systems framework. The present model advances beyond description to propose testable hypotheses about sequencing (stabilization before perturbation), timing (critical windows of plasticity), and consolidation (integration as mechanism of durability), claims that can be empirically evaluated through pragmatic trials comparing sequenced vs. concurrent interventions, or through longitudinal network analyses of symptom dynamics.

These developments support a view of psychiatric illness and recovery as emergent properties of complex systems. From this perspective, clinical care is less about correcting isolated dysfunctions and more about identifying constraints, modulating system stability, and supporting conditions under which adaptive reorganization can occur.

## 4 Psychedelics as controlled perturbations of complex systems

Psychedelic compounds can be understood as controlled perturbations of entrenched neural and psychological patterns rather than as agents that directly correct discrete molecular abnormalities. Neuroimaging studies consistently demonstrate that psychedelic states are associated with alterations in large-scale brain network organization, including decreased integrity of the default mode network and increased global connectivity (27). These changes are thought to transiently reduce the stability of rigid patterns of activity that underlie persistent symptoms.

The Relaxed Beliefs Under Psychedelics (REBUS) model situates these findings within a predictive processing framework, proposing that psychedelics reduce the precision-weighting of high-level priors that normally constrain perception, cognition, and affect (27).As these constraints loosen, previously suppressed or inaccessible information may enter awareness, increasing psychological flexibility and enabling alternative interpretations of experience. In systems terms, this reflects a temporary shift away from highly stable attractor states toward a more flexible and exploratory mode of organization.

This process can be understood as a form of perturbation: a time-limited destabilization of established patterns. Importantly, the therapeutic effect does not arise from disruption alone, but from what follows. As the system reorganizes, new patterns of thought, emotion, and behavior may emerge. Whether these changes persist depends on subsequent consolidation processes, including relational support, behavioral reinforcement, and integration of new meaning. These processes are not unique to psychedelic interventions but may be rendered more observable within them due to their intensity and temporal compression (47).

Comparable perturbation-reorganization dynamics are observed in other interventions (62). Electroconvulsive therapy (ECT), for example, is associated with changes in hippocampal structure, functional connectivity, and network-level organization (34, 63, 64). However, the subjective and clinical expression of these changes differs from that observed with psychedelic interventions. ECT is often experienced primarily as a reduction in symptoms and may be accompanied by cognitive side effects, whereas psychedelic treatments more commonly involve meaning-rich experiences and shifts in self-perception (65). The differences both clinically and experientially warrant a separate consideration in treatment planning and research design. Recognizing these differences is important for conceptual clarity, even as underlying system-level processes may share common features.

Ketamine provides a further illustration. As an NMDA receptor antagonist, ketamine produces rapid antidepressant effects accompanied by transient disruptions of habitual cognitive and affective patterns (66). These effects are associated with increased synaptic plasticity and altered network dynamics, creating a window during which entrenched patterns may be more amenable to change (66, 67).

These findings support a systems-based interpretation in which psychedelic and related interventions function as catalysts for reorganization rather than as targeted corrective agents (27). Clinical outcomes reflect the interaction between a temporarily destabilized system and the relational, psychological, and contextual conditions that shape its reorganization. One method of framing these distinctions is the difference between suppression of symptoms with daily dosing and an evocative method which triggers deeper change from the organism.

**Table 1** situates common psychiatric interventions along a continuum from suppressive to evocative processes within a systems-based framework, organized by primary phase of action (stabilization, modulation, acute perturbation, or relational consolidation). Interventions are compared across mechanism, temporal profile, degree of contextual dependence, and integration requirements, highlighting differences not only in biological targets but in their potential to constrain versus facilitate system reorganization. The classification of interventions along the suppressive–evocative continuum is theoretical and interpretive, derived from the present systems-based model rather than established empirical categorization. As such, the continuum is intended as a heuristic framework to generate hypotheses, emphasizing the proposed role of therapeutic context and integration in shaping the durability and depth of clinical outcomes.

**Table 1:** Systems-based intervention continuum.

Intervention	Phase	Mechanism	Temporal	Context	Integration	Mode	Limitations
SSRIs	Stabilization	Monoamine	Weeks–months	Minimal	Unstructured	Suppressive	Relapse
ECT	Acute	Seizure	Weeks	Minimal	Rare	Suppressive	Cognitive
TMS	Modulation	Cortical	Weeks	Minimal	None	Suppressive	Modest
Spravato	Acute+stab	NMDA	Hours–days	Constrained	Minimal	Mixed	Cost
Ketamine	Acute	NMDA	Hours–days	Variable	Developing	Hybrid	Durability
TMS (pers.)	Targeted	Network	Weeks	Emerging	Emerging	Hybrid	Evidence
Neurofeedback	Learning	Conditioning	Weeks–months	Moderate	Embedded	Evocative	Variation
Psychotherapy	Gradual	Relational	Months–years	Inherent	Continuous	Evocative	Slow
MDMA	Relational	Monoamine+Oxy	Sessions	High	Extensive	Evocative	Regulation
Psilocybin	Acute+consol.	5HT2A	Sessions	Central	Extensive	Evocative	Scale

## 5 Challenging assumptions about dosing, duration, and mechanism

One of the most consistent observations in psychedelic research is the dissociation between drug exposure and clinical outcome. In contrast to conventional psychopharmacology, where sustained receptor occupancy is typically assumed to be necessary for therapeutic benefit, psychedelics can produce durable improvements following one or a few administrations (68–70). Similar patterns are less reliably observed with ketamine and electroconvulsive therapy, where brief interventions may lead to lasting changes in mood and functioning Perturbation-reorganization dynamics are also observed with ketamine and electroconvulsive therapy, though these interventions typically require repeated administration to maintain clinical benefit, in contrast to the sustained effects often observed with psychedelics (71).

This temporal pattern calls into question the assumption that psychiatric treatments must operate through continuous molecular modulation. Instead, it suggests that therapeutic effects may arise from time-limited changes in system dynamics that initiate downstream processes of reorganization. In this view, pharmacologic action serves as a trigger for plasticity rather than as a mechanism requiring sustained presence.

From a systems perspective, these findings can be understood in terms of stability and transition. Persistent symptoms may reflect highly stable configurations that resist change. Interventions such as psychedelics or ketamine may reduce the stability of these configurations, allowing the system to transition into alternative states. The durability of benefit then depends on whether new patterns are consolidated through behavior, relationships, and environmental context.

This framework also helps explain variability in outcomes. Differences in preparation, therapeutic support, and integration may significantly influence whether perturbation leads to adaptive reorganization or to a return to prior patterns. Set and setting, once considered secondary factors, are therefore central determinants of both safety and efficacy (72).

At the same time, this context dependence introduces methodological challenges. Expectancy effects, functional unblinding, and therapist influence complicate efforts to isolate drug-specific mechanisms (73–75). These factors do not negate observed outcomes but indicate that therapeutic effects likely arise from interacting pharmacologic, psychological, and contextual processes rather than from any single component alone (76).

Considered together, these observations support a shift in emphasis from dose and duration to timing, context, and integration. Psychiatric interventions may be most effective not when continuously applied, but when introduced in a manner that enables systems to reorganize and sustain new patterns over time.

## 6 Psychological flexibility, meaning, and context dependence

Clinical outcomes following psychedelic therapy correlate only modestly with pharmacologic dose or acute subjective intensity. Instead, long-term benefit appears more closely associated with downstream changes in psychological flexibility, emotional processing, and meaning making (77–79).

These associations can be understood within a systems framework as reflecting shifts in how information is processed and integrated across levels. Increased psychological flexibility allows previously rigid interpretations and behavioral patterns to be updated in response to new information, while changes in meaning-making may alter the perceived significance of internal and external experiences. Together, these processes support transitions away from stable but maladaptive configurations toward more adaptive patterns of organization.

These findings align with broader evidence that meaning, expectation, and interpretive frameworks shape emotional and physiological regulation (80, 81). Psychedelics amplify these dynamics by temporarily altering salience hierarchies and increasing openness to new interpretations (27). Set and setting, once regarded as peripheral, are now recognized as central determinants of both safety and efficacy (82, 83).

This context sensitivity has important implications for interpretation and study design. Because therapeutic effects emerge from interacting pharmacologic and contextual variables, expectancy effects, functional unblinding, and therapist influence complicate efforts to isolate discrete causal mechanisms (75, 76). Rather than invalidating observed outcomes, these challenges suggest the need for pragmatic and hybrid trial designs that explicitly examine medication-therapy interactions and their contribution to durable change (84).

## 7 Ethical implications of destabilization

The same properties that confer therapeutic potential, heightened emotional permeability, identity lability, and openness to influence, also necessitate expanded ethical frameworks. Within a systems perspective, this heightened suggestibility reflects a temporary increase in sensitivity to relational and contextual input during periods of reduced system stability (85). While this creates therapeutic opportunity, it also introduces risk, as external influences may more readily shape emerging patterns of interpretation, behavior, and identity. Conventional consent models focused primarily on acute physiological risk are therefore insufficient for interventions capable of reshaping core beliefs, values, and relational patterns (86).

Psychedelics redistribute clinical power, shifting authority away from the prescriber toward the patient’s intrinsic regulatory and meaning-making capacities. This redistribution alters the clinician’s role from that of an expert interpreter toward a steward of safety, containment, and ethical restraint. In this context, a non-directive stance and clinical humility are not stylistic preferences but essential safeguards (87).

These dynamics also challenge institutional assumptions about psychiatric time and care delivery. Brief interventions capable of catalyzing nonlinear change strain reimbursement structures and training models optimized for chronic treatment and protocolized care (88). Evaluating safety, efficacy, and cost-effectiveness therefore requires extended temporal horizons and outcome measures that capture functional adaptation rather than short-term symptom change alone.

These considerations imply that ethical practice requires not only adherence to procedural safeguards, but active attention to how relational, environmental, and interpretive factors influence system reorganization over time (89).

## 8 Safety and risk considerations

Despite growing enthusiasm, psychedelic therapies are not without risk. Controlled clinical trials generally report low rates of serious adverse events; however, anxiety, confusion, transient dysphoria, and challenging psychological experiences are not uncommon during acute and subacute phases of treatment (68, 90). These reactions are typically self-limited within structured clinical settings but nonetheless represent predictable sources of distress that require preparation and monitoring (91).

Beyond controlled trials, naturalistic and post-trial data indicate that a subset of individuals experience prolonged psychological difficulties following psychedelic exposure, including persistent distress, perceptual disturbances, or functional impairment (92). Such outcomes appear more frequently outside highly controlled research environments and underscore the limits of extrapolating trial safety profiles to broader or less supervised populations.

Together, these findings highlight the importance of careful participant selection, thorough preparation, and longitudinal follow-up when translating psychedelic interventions beyond research contexts. They also caution against assuming that favorable safety profiles observed in specialized trials will necessarily generalize to routine clinical practice without equivalent levels of support and oversight (93). This framing reinforces the need for conservative implementation strategies that prioritize safety, clinician training, and contextual integrity over rapid dissemination. These observations also suggest the need for more refined assessment approaches capable of distinguishing between systems likely to undergo adaptive reorganization and those at risk for sustained destabilization or chaotic disruption following perturbation.

## 9 Assessment in systems-based psychiatry

A systems-based psychiatry requires a corresponding evolution in clinical assessment. Traditional diagnostic frameworks, while useful for classification and communication, are limited in their ability to capture the dynamic, multilevel organization of psychiatric illness (20). Categorical diagnoses often describe symptom clusters but provide limited insight into the underlying structure, stability, and adaptive capacity of the system as a whole (94).

Within a systems framework, assessment extends beyond diagnosis to include the identification of constraints and resources across interacting domains. These domains may include biological regulation, cognitive and emotional processing, developmental history, relational context, and environmental load. Rather than treating these factors as independent contributors, a systems approach evaluates how they interact to produce relatively stable patterns of functioning over time (95).

This perspective aligns with dimensional and network-based approaches to psychopathology, which conceptualize symptoms as mutually reinforcing elements within dynamic systems rather than as downstream expressions of a single latent disease entity. Such models emphasize patterns of connectivity, feedback loops, and system-level organization, offering a more flexible framework for understanding both stability and change (32).

Assessment in this context also involves evaluating system stability and flexibility. Highly rigid systems may be resistant to change but stable, whereas highly unstable systems may be prone to chaotic responses when perturbed. Clinical judgment must therefore consider not only the presence of symptoms but the system’s capacity to tolerate destabilization and reorganize adaptively (20, 26). This distinction has direct implications for treatment selection, timing, and intensity.

In practical terms, a systems-based assessment may integrate multiple sources of data, including clinical interview, psychometric measures, physiological indicators, and contextual information. The goal is not to replace existing diagnostic tools, but to situate them within a broader framework that captures system-level dynamics (96). Such an approach supports more individualized and context-sensitive treatment planning, particularly for interventions that intentionally modulate system stability.

These considerations suggest that advances in treatment must be accompanied by parallel advances in assessment. As psychiatric interventions increasingly target system-level processes, the ability to evaluate patterns of organization, constraint, and adaptive capacity will become central to safe and effective clinical care (97).

## 10 Sequencing and timing of interventions

A systems-based framework suggests that clinical effectiveness depends not only on the selection of interventions, but on their sequencing and timing within the broader trajectory of system change. Traditional psychiatric models often conceptualize treatment as continuous symptom suppression, whereas a systems perspective emphasizes phases of stabilization, perturbation, and consolidation (98).

Stabilizing interventions, including pharmacotherapy, behavioral regulation, and environmental support, may be necessary to reduce acute risk and establish sufficient system coherence prior to any destabilizing intervention. In contrast, perturbational interventions such as psychedelics, ketamine, or other plasticity-enhancing treatments may be most effective when introduced into systems that possess enough baseline stability to tolerate disruption without devolving into chaotic or maladaptive reorganization (33).

This sequencing reflects an underlying principle: interventions that increase plasticity are not inherently therapeutic in isolation but derive their benefit from the context in which they are delivered and the processes that follow (99, 100). A destabilized system may reorganize adaptively, regress to prior patterns, or shift toward less functional configurations depending on the relational, psychological, and environmental conditions present during and after the perturbation (100).

From this perspective, the concept of a “plasticity window” becomes clinically relevant (100). Periods of increased neural and psychological flexibility may represent time-limited opportunities during which targeted therapeutic input—such as psychotherapy, behavioral change, or relational repair, can have disproportionate impact on long-term outcomes (100, 101). The effectiveness of treatment therefore depends not only on what is delivered, but when it is delivered relative to system dynamics (101).

These concepts challenge models of care that prioritize isolated interventions without regard to temporal context. Instead, they support a phased approach in which stabilization, destabilization, and integration are deliberately coordinated. Such sequencing may improve both efficacy and safety, particularly for interventions capable of producing rapid or nonlinear change (102).

## 11 Integration and consolidation

If perturbation initiates the process of change, integration determines its durability. Within a systems framework, integration refers to the processes through which newly emerging patterns of thought, emotion, and behavior are stabilized and incorporated into ongoing functioning. Without effective integration, systems are likely to revert toward prior attractor states, particularly when those states have been reinforced over extended periods (20).

Integration is not a single event but an extended process involving multiple domains. Behavioral changes, relational experiences, and shifts in meaning all contribute to the consolidation of new patterns. Therapeutic support during this phase may include psychotherapy, reflective practices, social reinforcement, and environmental modifications that align with and strengthen emerging configurations (103).

This process can be understood as the gradual re-establishment of system stability around a new configuration. Whereas perturbation temporarily reduces stability, integration rebuilds it in a manner that supports adaptive functioning. The durability of clinical benefit therefore depends less on the intensity of the initial intervention and more on the consistency and alignment of post-intervention processes (104).

Importantly, integration also provides a mechanism for translating transient experiential insights into sustained behavioral change. Experiences of increased connectedness, emotional release, or altered self-perception, commonly reported following psychedelic interventions, do not automatically lead to lasting improvement. Their clinical significance depends on whether they are meaningfully incorporated into the individual’s ongoing patterns of thought, behavior, and relationship (105).

These observations reinforce the view that psychiatric interventions are best understood as components of a broader temporal process rather than as discrete events. The clinical task extends beyond initiating change to supporting the conditions under which that change can be maintained (76). In this sense, integration is not ancillary to treatment, it is central to its success (76, 105).

## 12 Clinical implementation considerations

The translation of systems-based principles into clinical practice requires careful attention to structure, workflow, and continuity of care. Traditional psychiatric models, often organized around brief, episodic encounters, are not well suited to interventions that depend on temporal sequencing, relational context, and longitudinal integration (46, 106). A systems-based approach therefore implies not only new treatments, but new models of care delivery.

Central to this shift is the recognition that interventions capable of modulating system stability, particularly those that increase plasticity, must be embedded within broader care frameworks that include preparation, monitoring, and integration. These elements are not ancillary but integral to both safety and efficacy. Preparation establishes the psychological and relational conditions necessary for adaptive perturbation, while ongoing monitoring ensures that destabilization remains within a tolerable range (107). Integration then consolidates emerging changes into durable patterns of functioning.

Such an approach is inherently interdisciplinary. Effective implementation requires coordinated contributions from psychiatry, psychotherapy, and other supportive modalities, including behavioral and social interventions. The psychiatrist retains a central role in assessment, risk stratification, and medical oversight, but therapeutic outcomes depend on collaboration across disciplines and over time (46, 107).

Scalability presents an additional challenge. Intensive, highly supported models of care may be difficult to extend to broader populations without adaptation (106). Task-sharing approaches—where specific components of care are delivered by providers with varying levels of specialization—offer one potential solution. In such models, non-specialist clinicians may deliver structured preparation and integration support, while psychiatrists and other specialists focus on assessment, supervision, and the management of higher-risk interventions (108).

Despite growing clinical and scientific interest, the implementation of psychedelic therapies remains highly heterogeneous across regulatory jurisdictions. In the United States, classic psychedelics such as psilocybin remain classified as Schedule I substances at the federal level (109), and the U.S. Food and Drug Administration’s 2024 decision not to approve MDMA-assisted therapy for PTSD highlighted ongoing concerns regarding trial methodology, functional unblinding, therapist effects, and implementation standards (110). At the same time, federal policy continues to evolve. A recent U.S. Presidential Executive Order directed federal agencies to explore mechanisms for accelerating research and investigational access pathways for emerging treatments targeting serious mental illness, including psychedelic compounds (111)(Executive Order No. 14401, 2026). Regulatory pathways are also evolving differently elsewhere. Australia’s Therapeutic Goods Administration (TGA) has authorized limited psychiatrist prescribing of psilocybin and MDMA under controlled conditions (112), while several European countries continue to expand regulated clinical trial and compassionate-access frameworks for psychedelic-assisted therapies (113). These divergent trajectories underscore that the translation of psychedelic therapies into clinical practice will likely proceed unevenly across jurisdictions and will depend not only on pharmacologic evidence, but also on the development of appropriate training standards, infrastructure, longitudinal safety monitoring, and context-sensitive models of care. From a systems-based perspective, these regulatory differences are not peripheral considerations but part of the broader relational and institutional context that shapes implementation, safety, and long-term clinical outcomes.

These considerations suggest that implementation should proceed cautiously, prioritizing fidelity to core principles over rapid expansion. Systems-based care depends not only on the availability of specific interventions, but on the integrity of the relational, organizational, and temporal structures within which those interventions are delivered.

## 13 Workforce and training implications

The adoption of a systems-based psychiatry carries significant implications for workforce development and professional training. Traditional psychiatric education has emphasized diagnostic classification and pharmacologic management, with comparatively less focus on relational processes, developmental context, and longitudinal system dynamics (95, 114). While this approach aligns with mechanistic models of care, it is less well suited to interventions that depend on timing, context, and integration for their effects.

A systems-based framework requires a broader set of competencies. Clinicians must be able to assess risk and adaptive capacity across multiple domains, including biological regulation, psychological flexibility, developmental history, and relational context (36). This includes recognizing when a system is sufficiently stable to tolerate perturbation, when preparatory work is required, and when destabilizing interventions may increase risk. Such judgments cannot be derived from protocols alone and depend on clinical experience, supervision, and longitudinal understanding of the patient (115).

These demands highlight the importance of interdisciplinary care. Psychiatric treatment in this model is not confined to medication management but involves coordinated contributions from psychotherapy, social context, and behavioral interventions (114). Psychiatrists retain a central role in risk assessment, medical oversight, and the stewardship of interventions that alter system stability, but effective care depends on collaboration across disciplines. Within this framework, psychotherapeutic, developmental, and social expertise are not ancillary to biological psychiatry but represent equal and essential contributors to both understanding and treating psychiatric illness (116).

Training models must therefore evolve to reflect these realities. In addition to pharmacology and diagnosis, education should include developmental psychopathology, trauma-informed care, relational dynamics, and the ethical management of interventions that increase psychological plasticity (117). Supervised clinical experience and ongoing consultation are essential for developing the capacity to work with complex, nonlinear change processes. Short-term certification models alone are unlikely to provide sufficient preparation for these demands (114, 117).

At the same time, scalability requires flexibility. Systems-based care is compatible with tiered and task-sharing models in which different providers operate within defined scopes of practice (118, 119). Non-specialist clinicians may deliver structured preparation and integration support, while psychiatrists and other specialists retain responsibility for assessment, risk stratification, and oversight of destabilizing interventions. Such models align with broader global mental health strategies that emphasize efficient use of limited specialist resources while maintaining safety and continuity of care (120).

This material suggests that the transition toward a systems-based psychiatry will depend not only on new treatments, but on corresponding changes in training, supervision, and collaborative care structures. The capacity to safely introduce and integrate interventions that alter system stability is a learned clinical skill, requiring time, experience, and interdisciplinary support (119, 120).

## 14 Methodological challenges and research implications

Despite growing interest in systems-based interpretations of psychiatric change, several methodological challenges constrain current evidence and warrant careful consideration in the design of future research. Psychedelic trials face particular difficulties with causal inference: expectancy effects, functional unblinding, and therapist influence are difficult to eliminate in studies involving highly salient subjective experiences and may inflate effect sizes or obscure the relative contribution of pharmacologic versus contextual mechanisms (73, 75, 76). These challenges are compounded by the fact that many trials involve highly selected samples, intensive preparatory support, and extensive follow-up that may not generalize to routine clinical practice (121). While such designs are appropriate for early-phase safety and efficacy assessment, they limit conclusions about scalability and real-world effectiveness.

The gap between controlled research settings and clinical environments is not merely logistical but conceptual. From a systems perspective, the relational, environmental, and contextual conditions that surround an intervention are not confounds to be eliminated but active contributors to outcome (82, 83, 107). Real-world implementation introduces variability in patient populations, provider training, and therapeutic context: factors that a systems-based framework treats as central determinants of whether perturbation leads to adaptive reorganization or regression toward prior patterns. Without implementation models that preserve key elements of preparation, support, and integration while adapting to the realities of clinical practice, there is a meaningful risk that outcomes observed in controlled trials will not generalize, potentially leading to reduced efficacy or increased adverse events (106, 122, 123).

These considerations point toward the need for innovation in research design. Traditional randomized controlled trials are optimized for isolating single variables but may be less well suited to evaluating interventions whose effects arise from interactions across multiple domains. Pragmatic and hybrid designs, including comparative effectiveness trials, adaptive designs, and longitudinal studies assessing outcomes over extended time horizons, may more closely approximate real-world conditions while maintaining methodological rigor (35, 124). Outcome measures should expand beyond short-term symptom reduction to include functional capacity, relational dynamics, and meaning-based constructs, which may better capture the types of change reported in psychedelic and other systems-oriented interventions (125).

Critically, future studies should be designed not to isolate drug effects entirely from therapeutic context, but to explicitly examine how pharmacologic interventions, psychotherapy, and environmental factors jointly contribute to outcomes. A systems-based framework accommodates these methodological uncertainties by resisting reductive explanations and emphasizing probabilistic rather than deterministic claims, an orientation that applies equally to psychotherapy, neuromodulation, and other complex interventions where blinding is imperfect and outcomes depend on relational and contextual variables (48, 126, 127).

## 15 Toward a systems-based psychiatry

Taken together, these findings support a broader reconceptualization of psychiatric illness and treatment. A systems-based psychiatry views mental disorders not as discrete disease entities arising from isolated biological defects, but as patterns of constrained organization emerging from interactions across neural, psychological, relational, and environmental domains (128).

Within this framework, treatment is understood as the strategic modulation of system stability and plasticity over time. Stabilizing interventions may be used to reduce acute risk and establish coherence, while destabilizing interventions may create opportunities for reorganization. The effectiveness of care depends on how these phases are sequenced and integrated within a broader therapeutic process (20).

This model does not reject existing approaches but rather situates them within a more comprehensive framework that emphasizes interaction, timing, and context. Pharmacologic treatments, psychotherapies, and social interventions can all be understood as inputs into a dynamic system, with outcomes determined by their interaction rather than by any single component alone (129).

A systems-based perspective also encourages a shift in clinical focus from symptom suppression to the promotion of adaptive capacity. This includes enhancing flexibility, supporting relational functioning, and facilitating the integration of new patterns of meaning and behavior. Such an approach aligns more closely with the lived experience of patients and may better account for the variability and complexity observed in clinical practice (46).

## 16 Conclusion

Psychiatry stands at a moment of conceptual transition. Mechanistic models have produced significant advances—from receptor-based pharmacology to circuit-based neuromodulation and emerging precision approaches. Yet a persistent gap remains between the explanatory power of current models and the complexity, variability, and context dependence observed in clinical practice. This gap reflects not a failure of these models, but the limits of frameworks that privilege linear causation over dynamic system behavior.

This is not a new observation. The biopsychosocial model, RDoC, The Hierarchical Taxonomy of Psychopathology (HiTOP) and network theories of psychopathology have each moved the field toward cross-domain thinking (130). A systems-based psychiatry builds on these efforts but argues that integration alone is insufficient without a corresponding shift in how change itself is modeled—foregrounding the nonlinear, emergent, and time-dependent dynamics that characterize transitions between health and illness. Concepts from complexity science, including attractor states, critical transitions, and dynamic systems modeling, now offer testable frameworks for studying these processes. The central task is no longer conceptual, but operational: developing reliable measures of system properties such as flexibility, constraint, and adaptive capacity that can be deployed in clinical research and care.

Psychedelic-assisted therapies offer a particularly revealing case. Like many psychiatric interventions, their outcomes are shaped by context, expectation, and integration. What distinguishes them is that they reliably induce transient destabilization of established patterns—followed, in supportive contexts, by reorganization into more adaptive configurations. In this sense, they make visible a generalizable mechanism of therapeutic change: perturbation of rigid system states, increased flexibility or entropy, and subsequent restabilization at a higher level of organization. While the evidence base remains early and requires continued rigor, this pattern is consistent with broader observations across psychotherapy, neuromodulation, and behavioral interventions.

From this perspective, treatment becomes the strategic modulation of system states through timing, sequencing, and context. Variability in outcomes is therefore not simply noise, but a reflection of underlying system dynamics and initial conditions. Recovery, accordingly, can be reframed—not as symptom reduction alone, but as the restoration of flexibility, coherence, and adaptive functioning across domains. This formulation helps explain why symptomatic remission and functional recovery often diverge, and why durable change depends on processes that extend beyond the acute intervention itself.

The path forward is not a binary choice between mechanistic and systems-based approaches, but a reorganization of existing knowledge within a framework that accommodates context, emergence, and longitudinal change alongside mechanism and specificity. If developed with empirical discipline, such a framework would enable earlier identification of dysregulation, provide a principled basis for prevention, and support integrative models of care that target system-level health rather than isolated symptoms. Psychiatry, in this view, is not only the treatment of disorder, but the stewardship of conditions under which adaptive organization and what we call healing can reliably emerge and be sustained.

## Data Availability

The original contributions presented in the study are included in the article/**Supplementary Material**. Further inquiries can be directed to the corresponding author.
