# Supplementary material for: Systems-based psychiatry: insights from psychedelic research on mechanisms of healing
Source: Front Psychiatry. 2026 Jul 13;17:1789902. doi: 10.3389/fpsyt.2026.1789902 (PMC13403111; doi:10.3389/fpsyt.2026.1789902)
Supplement: Supplementary file 1 [file SupplementaryFile1.docx]

**I. Multiaxial Systems Assessment**

**Purpose**

The Multiaxial Systems Assessment translates the systems-based model of psychiatric healing into a practical clinical formulation tool. It is not intended to replace DSM diagnosis, validated symptom measures, or evidence-based treatment planning. Rather, it organizes these elements within a broader clinical logic that reflects how psychiatric suffering and recovery unfold across biological, psychological, relational, contextual, and existential domains.

Conventional psychiatric assessment often begins by asking, “What diagnosis best explains this presentation?” A systems-based assessment asks a complementary question: “What is maintaining this person’s current state, what capacities remain available for change, and what can the system safely tolerate next?” This distinction is particularly important when considering intensive, experiential, or destabilizing interventions, where clinical judgment must include not only potential efficacy, but also readiness, stability, support, and capacity for integration.

The assessment is organized around four core axes: **Quality of Life, Body, Mind, and Spirit**. These axes are linked by a cross-cutting dimension of **Regulation/Adaptability**, which evaluates the system’s capacity to maintain stability while tolerating change. Together, these domains support a required **phase-of-care determination**: Stabilization, Foundation Building, Perturbation-Ready, Active Processing, Integration, or Maintenance.

**Core Axes**

**Axis 1: Quality of Life / Function**

Quality of life and functional capacity are treated as the primary outcome axis. Symptom reduction remains important, but clinical success is anchored in whether the person’s lived experience improves across daily functioning, relationships, vitality, role performance, and sense of agency.

**Key constructs**

- Overall quality of life
- Physical, emotional, social, and role functioning
- Fatigue, sleep, pain, and vitality
- Functional impairment and disability
- Patient-defined goals for meaningful improvement

**Commonly used tools**

- PROMIS-29 or PROMIS Global Health
- WHO Disability Assessment Schedule 2.0
- Sheehan Disability Scale
- Clinical assessment of occupational, relational, and daily functioning
- Patient-defined quality-of-life markers

**Axis 2: Body / Biological Substrate**

The Body axis assesses physiological factors that may constrain or support psychiatric recovery. These factors are not treated as isolated biological defects, but as elements of the larger system that can affect mood, cognition, arousal, flexibility, and treatment responsiveness.

**Key constructs**

- Sleep–wake stability
- Autonomic and stress regulation
- Inflammation, metabolic status, endocrine function, and nutritional sufficiency
- Medication effects on vitality, cognition, and affect
- Medical comorbidity burden
- Neurophysiological patterns, when clinically indicated

**Commonly used tools**

- Sleep history and sleep diaries
- Insomnia Severity Index
- Epworth Sleepiness Scale
- Vital signs, BMI, and metabolic labs as indicated
- Medication side-effect review
- Targeted laboratory assessment when clinically indicated
- qEEG or other neurophysiological assessment when clinically appropriate

**Axis 3: Mind / Psychological Patterning**

The Mind axis includes DSM-relevant symptom patterns while also assessing psychological organization, cognitive and emotional flexibility, avoidance, rumination, reflective capacity, and dominant coping patterns. Diagnosis is retained, but it is placed within a broader formulation of psychological patterning and adaptive capacity.

**Key constructs**

- Symptom burden
- Cognitive and emotional flexibility
- Rumination, avoidance, threat sensitivity, and hypervigilance
- Reflective capacity and distress tolerance
- Dissociation, compulsivity, and substance-mediated regulation
- Dominant DSM-relevant patterns

**Commonly used tools**

- PHQ-9
- GAD-7
- PTSD Checklist for DSM-5
- Yale-Brown Obsessive Compulsive Scale
- Acceptance and Action Questionnaire–II
- Ruminative Response Scale
- Dissociative Experiences Scale-II when indicated
- AUDIT and DAST when indicated
- Clinical assessment of mentalization, self-observation, and affect regulation

**Axis 4: Spirit / Meaning, Connection, and Coherence**

The Spirit axis assesses meaning, purpose, existential orientation, and connectedness. It does not evaluate the presence or absence of religious belief. Rather, it asks whether the person has access to coherent sources of meaning, value, relational connection, and orientation that can support recovery and integration.

This axis is particularly relevant in transformative or psychedelic-assisted work, where experiences may alter identity, values, relational patterns, and existential understanding.

**Key constructs**

- Meaning and purpose
- Connection to self, others, community, nature, or the larger world
- Existential coherence and values orientation
- Moral injury or spiritual conflict
- Identity coherence
- Capacity to integrate insight into life direction

**Commonly used tools**

- Watts Connectedness Scale
- FACIT-Sp-12
- Values-based clinical interview
- Assessment of moral injury, existential distress, or spiritual conflict when relevant
- Clinical exploration of meaning, identity, belonging, and purpose

**Cross-Cutting Dimension: Regulation / Adaptability**

Across all four axes, clinicians assess the patient’s capacity for regulation and adaptive change. This dimension is central to clinical decision-making because the same intervention may be helpful, ineffective, or destabilizing depending on the patient’s current level of stability and flexibility.

The patient’s system state may be described as:

- **Rigid / over-controlled:** organized but inflexible, defended, constricted, repetitive, or difficult to shift
- **Stable / adaptive:** sufficiently regulated, flexible, reflective, and able to use support
- **Unstable / chaotic:** poorly regulated, overwhelmed, unsafe, or prone to fragmentation

Capacity for change may be rated as **low, moderate, or high**.

**Psychological Capacities and Resources**

Because treatment sequencing depends not only on symptom severity but also on functional capacity, the assessment should include a brief clinician-rated estimate of psychological capacities and resources.

**Suggested domains**

- Reflective capacity
- Distress tolerance
- Attachment security or ability to use support
- Cognitive and emotional flexibility
- Capacity for self-observation

These capacities help determine whether the patient is ready for deeper or destabilizing work, whether Foundation Building is needed first, or whether Stabilization should be prioritized.

**Decision Modifiers**

Decision modifiers are factors that alter treatment thresholds, pacing, safety, or level of care. They are not separate axes, but clinically important constraints that may amplify risk or reduce adaptive capacity across the system.

**Common decision modifiers include:**

- High developmental burden or adverse childhood experiences
- Active environmental stress, threat, or instability
- Addictive regulation patterns
- Dissociation risk
- Bipolar or psychosis vulnerability
- Significant medical or metabolic burden
- Limited relational support
- Unsafe or unpredictable living conditions

These modifiers are evaluated not only for their direct effects, but also for how they propagate dysregulation across domains.

**Required Clinical Output: Phase-of-Care Determination**

The Multiaxial Systems Assessment should conclude with a phase-of-care determination. This required output links assessment directly to treatment sequencing.

The phase-of-care determination should identify the patient’s current clinical position as one of the following:

- **Stabilization:** safety, containment, and basic regulation are the immediate priorities
- **Foundation Building:** capacities, supports, and physiological stability must be strengthened before deeper work
- **Perturbation-Ready:** the system appears stable enough to tolerate deeper or potentially destabilizing intervention
- **Active Processing:** the patient is engaged in intensive therapeutic work or active reorganization
- **Integration:** insights or symptom shifts need to be translated into behavior, identity, relationships, and function
- **Maintenance:** gains are sufficiently stable and the focus is relapse prevention, flexibility, and continued development

This determination should be based on quality-of-life impairment, biological burden, psychological organization, meaning and coherence, regulation/adaptability, psychological capacities, and decision modifiers.

**Clinical Reframe**

Rather than asking only, “What diagnosis explains this presentation?” the Multiaxial Systems Assessment asks:

- Which axes are most impaired?
- Which systems are rigid, overwhelmed, or unstable?
- Which capacities remain available for change?
- What constraints reduce adaptive capacity?
- What phase of care does this person need now?
- What can the system safely tolerate next?

**II. Readiness, Regulation, and Rigidity Mapping**

**Purpose**

Readiness, Regulation, and Rigidity Mapping determines whether a patient can safely engage in intensive, destabilizing, or transformative interventions, or whether Stabilization or Foundation Building is required first. In a systems-based model, such interventions are understood as perturbations: time-limited disruptions that may loosen maladaptive patterns, increase flexibility, and create an opportunity for reorganization.

This section applies the axes and constructs defined in Section I to a specific clinical question:

**Is this patient stable enough to reorganize, or are they currently too unstable, unsupported, rigid, or overwhelmed to benefit safely from perturbation-oriented work?**

Perturbation-oriented interventions may include trauma-focused psychotherapy, EMDR, ketamine-assisted psychotherapy, psychedelic-assisted therapy where legal, intensive meditation or retreat-based work, and other experiential or biologically disruptive treatments.

**Core Determinants of Readiness**

Readiness should be judged across the four axes described in Section I, with particular attention to:

- **Quality of Life / Function:** severity of impairment, functional collapse, role disruption, and patient-defined need for meaningful change
- **Body / Biological Substrate:** sleep stability, medical risk, medication burden, substance use, autonomic regulation, and physiological resilience
- **Mind / Psychological Patterning:** distress tolerance, reflective capacity, flexibility, dissociation risk, impulse control, reality testing, and suicidality
- **Spirit / Meaning, Connection, and Coherence:** existential stability, identity coherence, moral injury or spiritual conflict, connectedness, and capacity to translate insight into life direction
- **Regulation / Adaptability:** whether the system is rigid, stable-adaptive, or unstable-chaotic
- **Psychological Capacities:** whether the patient can observe, tolerate, and integrate difficult internal experience
- **Decision Modifiers:** contextual, developmental, medical, or psychiatric risks that alter pacing, intensity, and level of care

Readiness is not determined by motivation alone. A patient may strongly desire intensive treatment but still require preparation if adaptive capacity is low or decision modifiers are high.

**Readiness Ratings**

Using the definitions in Section I, clinicians should rate the following domains:

**Primary system state:**
Rigid / Stable-Adaptive / Unstable-Chaotic

**Capacity for change:**
Low / Moderate / High

**Quality-of-life impairment:**
Minimal / Mild / Moderate / Severe

**Biological constraint:**
Low / Moderate / High

**Psychological capacity:**
Low / Moderate / High

**Meaning/coherence:**
Intact / Fragile / Absent

**Decision modifier severity:**
Minimal / Moderate / High

**Primary constraints on adaptive capacity:**

**Stabilization-First Conditions**

Perturbation-oriented interventions should generally be deferred when any of the following are present:

- Active suicidal intent or recent suicide attempt
- Acute psychosis with impaired reality testing
- Mania or unstable bipolar disorder
- Severe dissociation with loss of function
- Active substance withdrawal or uncontrolled dependence
- Unsafe or actively traumatic environment
- Severe medical instability
- Inability to maintain basic treatment agreements or follow-up

In these situations, the appropriate phase of care is usually **Stabilization**.

**Conditions Requiring Preparation**

Perturbation-oriented work may be possible later, but additional preparation is usually needed when the following are present:

- History of psychosis or bipolar disorder
- Moderate dissociation risk
- Severe developmental trauma burden
- Recent major loss or trauma
- Limited relational support
- Low distress tolerance
- High avoidance, compulsive control, or addictive regulation
- Significant medication complexity
- Medical comorbidity requiring coordination
- Fragile therapeutic alliance

In these situations, the appropriate phase of care is usually **Foundation Building**.

**Minimum Indicators of Readiness**

Minimum indicators of readiness for perturbation-oriented work include:

- No acute suicidal intent, mania, psychosis, or uncontrolled substance withdrawal
- Sufficient symptom stability over time
- Safe and predictable living situation
- Adequate sleep or active plan to improve sleep stability
- At least one reliable support relationship
- Working therapeutic alliance
- Capacity for reflection and self-observation
- Capacity to tolerate affective activation without fragmentation
- Motivation that extends beyond immediate symptom suppression
- Willingness to engage in preparation, integration, and follow-up care

**Interpretation Patterns**

**Diffuse Rigidity**

Rigidity or constraint is present across multiple axes, such as physiological dysregulation, psychological constriction, relational narrowing, and loss of meaning.

**Clinical implication:**
A phased, multimodal approach is usually indicated. Treatment should first address the constraint that most limits adaptive capacity.

**Focal Rigidity**

One axis appears especially constrained while other capacities remain intact.

**Clinical implication:**
Targeted intervention may be appropriate. Examples include primary sleep disruption, trauma avoidance, relational conflict, or meaning collapse.

**High Distress with High Instability**

The patient is suffering intensely but lacks sufficient regulation, containment, or self-observation.

**Clinical implication:**
Prioritize Stabilization and Foundation Building. Perturbation-oriented work should generally be deferred.

**Moderate Rigidity with Adequate Stability**

The patient is stuck but retains enough regulation, support, and reflective capacity to tolerate change.

**Clinical implication:**
This may represent an optimal window for perturbation-oriented or deeper therapeutic work.

**Improved Symptoms with Persistent Functional or Meaning Impairment**

Symptom scores may improve while quality of life, relational functioning, or existential coherence remain impaired.

**Clinical implication:**
Treatment should shift toward Integration, values-based action, relational repair, and functional restoration rather than stopping care prematurely.

**Required Clinical Output: Readiness Determination**

This assessment should conclude with a clear readiness determination:

- **Not currently appropriate:** Stabilization required
- **Possibly appropriate later:** Foundation Building required first
- **Appropriate with caution:** Proceed only with enhanced preparation, support, monitoring, or modified intensity
- **Appropriate:** Patient appears perturbation-ready with adequate preparation and follow-up

This readiness determination should directly inform the phase-of-care assignment:

- Stabilization
- Foundation Building
- Perturbation-Ready
- Active Processing
- Integration
- Maintenance

**Clinical Principle**

Destabilizing treatments are safest when the system is stable enough to reorganize rather than fragment. The goal is not to avoid perturbation, but to introduce it at the right time, at the right intensity, within sufficient biological, psychological, relational, contextual, and ethical containment.

**III. Intervention Sequencing Framework**

**Purpose**

The Intervention Sequencing Framework links multiaxial assessment to clinical action. Its purpose is to help clinicians determine not only what treatments may be appropriate, but when they should be introduced, what should be deferred, and how much intensity the patient’s current system can safely tolerate.

In a systems-based model, psychiatric interventions differ in their primary function. Some interventions stabilize the system, some build adaptive capacity, some perturb rigid patterns, some support active processing, and others consolidate or maintain gains. The same intervention may serve different functions depending on timing. Psychotherapy, for example, may function as stabilization, foundation building, active processing, or integration depending on the patient’s current phase of care.

The framework below is not a fixed protocol. It is a decision aid for matching treatment intensity to the patient’s phase of care, regulation/adaptability profile, psychological capacities, decision modifiers, and current constraints across the four axes described in Section I.

**Phase-of-Care Framework**

| **Phase** | **System State** | **Primary Aim** | **Representative Interventions** | **Clinical Caution** |
| --- | --- | --- | --- | --- |
| **Stabilization** | Unsafe, overwhelmed, chaotic, medically unstable, or unable to reliably use support | Restore safety, containment, physiological regulation, and basic functioning | Safety planning, higher level of care when indicated, supportive psychotherapy, medication stabilization, withdrawal management, case management, environmental stabilization | Defer perturbation-oriented work until basic regulation, follow-up, and safety are established |
| **Foundation Building** | Partially stable but under-resourced; limited distress tolerance, fragile alliance, poor sleep, weak support, or high decision-modifier burden | Increase adaptive capacity so future change is safer and more durable | CBT, DBT skills, ACT, trauma-informed supportive therapy, medication optimization, sleep/circadian stabilization, lifestyle interventions, mindfulness, relational support | Motivation for deeper work should not be mistaken for readiness; preparation is active treatment, not delay |
| **Perturbation-Ready** | Stable enough to tolerate activation, with persistent rigidity, treatment resistance, or constrained adaptive capacity | Introduce selected interventions that loosen maladaptive patterns and create opportunity for reorganization | EMDR, trauma-focused psychotherapy, ketamine-assisted psychotherapy, psychedelic-assisted therapy where legal, intensive experiential work, neuromodulation when indicated | Perturbation is an opportunity for reorganization, not a standalone cure; preparation, containment, and integration planning are essential |
| **Active Processing** | Engaged in intensive therapeutic change, trauma processing, altered-state work, or active reorganization | Maintain enough safety, orientation, and containment for new learning to emerge without fragmentation | Ongoing psychotherapy, carefully paced trauma processing, somatic regulation, post-session integration, values clarification, behavioral experiments, close follow-up | Intensity, insight, or emotional release should not be mistaken for durable change; monitor for destabilization or dependency |
| **Integration** | Insight, symptom shift, emotional release, or altered self-understanding has occurred but is not yet consolidated into life structure | Translate change into daily functioning, relationships, identity, values, and embodied action | Psychotherapy, behavioral activation, ACT and values-based action, relationship or family work, lifestyle restructuring, community or group integration support | The most common integration failure is insight without embodiment; assess whether behavior and function are actually changing |
| **Maintenance** | Gains are sufficiently stable; improved function, regulation, resilience, and flexibility are present | Preserve flexibility, prevent relapse, reduce unnecessary treatment intensity, and support continued development | Less frequent psychotherapy, medication review, self-directed practices, community engagement, relational and lifestyle supports, booster sessions when needed | Maintenance is not abandonment; monitor early shifts in sleep, function, relationships, meaning, and regulation |

**Using the Framework Clinically**

The phase-of-care designation should be revisited over time. Patients do not always move through these phases linearly. A patient may move from Active Processing back to Stabilization if delayed destabilization emerges, or from Maintenance back to Foundation Building after a major loss, illness, relapse, or environmental stressor. Systems-based care requires adaptive reassessment rather than adherence to a fixed sequence.

When choosing an intervention, clinicians should ask:

- Does this intervention primarily stabilize, build capacity, perturb, process, integrate, or maintain?
- Is the patient’s current regulation/adaptability profile compatible with this level of intensity?
- Which axis is most constraining adaptive capacity now?
- Are psychological capacities sufficient for the proposed intervention?
- Which decision modifiers require caution, delay, modification, or higher level of care?
- What needs to be in place for gains to consolidate into quality of life, function, relationship, body, mind, and meaning?

**Required Clinical Output**

The intervention sequencing process should conclude with a brief treatment-direction statement:

**Current phase of care:**
Stabilization / Foundation Building / Perturbation-Ready / Active Processing / Integration / Maintenance

**Primary treatment target:**
Quality of Life / Body / Mind / Spirit / Regulation-Adaptive Capacity / Contextual Constraint

**Primary intervention function:**
Stabilize / Build Capacity / Perturb / Process / Integrate / Maintain

**What should be done now:**

**What should be deferred:**

**What conditions would justify moving to the next phase:**

**Clinical Principle**

Treatment sequencing in systems-based psychiatry is not a linear march from diagnosis to intervention. It is an adaptive process of determining what the person needs next, what the system can safely tolerate now, and how therapeutic change can be consolidated into a more flexible, coherent, and functional life.

**IV. Integration and Long-Horizon Monitoring**

**Purpose**

Integration and long-horizon monitoring are essential to systems-based psychiatric care. Interventions that produce insight, emotional activation, symptom relief, altered states, or rapid shifts in self-understanding do not automatically produce durable change. The central clinical question after meaningful therapeutic movement is whether the change becomes embodied in quality of life, physiology, psychological patterning, relationships, meaning, and daily behavior.

Integration is therefore not limited to discussing a powerful experience. It is the process by which new learning becomes stabilized across the system. Long-horizon monitoring asks whether the patient is becoming more flexible, coherent, functional, and resilient over time, or whether delayed destabilization, dependency, avoidance, or functional decline is emerging.

**Integration Targets**

Using the axes described in Section I, clinicians should monitor whether gains are translating into:

- **Quality of Life / Function:** improved daily functioning, role performance, relationships, vitality, agency, and patient-defined markers of meaningful improvement
- **Body / Biological Substrate:** improved sleep, recovery, energy, autonomic regulation, medical stability, and reduced reliance on substance-mediated regulation
- **Mind / Psychological Patterning:** greater flexibility, reduced rumination or avoidance, improved self-observation, increased affect tolerance, and behavior change
- **Spirit / Meaning, Connection, and Coherence:** grounded meaning-making, values-based action, relational repair, increased connection, and coherent life direction

**Markers of Successful Integration**

Markers of successful integration include:

- Improved quality of life and daily functioning
- Better sleep, recovery, and physiological stability
- Increased emotional range without flooding
- Greater cognitive and behavioral flexibility
- Reduced avoidance, rumination, or compulsive control
- Improved relational repair and capacity for support
- Increased meaning, coherence, and values-based action
- Ability to tolerate uncertainty and stress without immediately returning to old patterns
- Stable engagement with treatment, community, and ordinary responsibilities

**Warning Signs of Incomplete Integration**

Integration should be reassessed when clinical change remains primarily experiential, verbal, or symbolic without corresponding behavioral or functional change.

Common warning signs include:

- Insight without behavioral change
- Symptom improvement without quality-of-life improvement
- Powerful experience followed by avoidance of ordinary responsibilities
- Premature discontinuation of care
- Increased isolation after treatment
- Idealization or devaluation of clinicians, treatments, groups, or prior life roles
- Repeated pursuit of destabilizing experiences without consolidation
- Substitution of spiritual or psychological language for concrete action
- Persistent relational conflict despite claimed transformation

**Serious Long-Horizon Risks**

Delayed adverse effects warrant the same clinical attention as acute adverse events. Some risks may emerge days, weeks, or months after an intensive intervention, especially when altered self-understanding, relational shifts, or destabilized defenses are not adequately integrated.

Serious concerns include:

- Emergent mania or hypomania
- Psychosis or impaired reality testing
- Increased suicidality or self-harm risk
- Severe dissociation, derealization, or depersonalization
- Persistent perceptual disturbances
- Functional decline
- Substance relapse or escalation
- Boundary disturbances, dependency, or coercive relational dynamics
- Intensified despair, nihilism, or existential disorganization
- Medical, sleep, or autonomic destabilization

When these concerns arise, the phase of care should be reassessed. A patient may need to move from Integration or Active Processing back to Stabilization or Foundation Building. Systems-based care is not strictly linear; it requires adaptive reassessment as the patient’s state changes.

**Maintenance and Relapse Prevention**

Maintenance is indicated when the patient has achieved improved function, greater flexibility, and sufficient stability. The goal is to preserve adaptive capacity while avoiding unnecessary treatment intensity.

Maintenance may include:

- Early warning sign identification
- Relapse prevention planning
- Periodic review of sleep, function, relationships, and meaning
- Medication review and tapering only when clinically appropriate
- Ongoing self-directed practices
- Community and relational support
- Booster psychotherapy or integration sessions when needed
- Reassessment after major stressors, losses, medical changes, or life transitions

**Required Clinical Output**

Integration and monitoring should conclude with a brief clinical determination:

**Are gains translating into quality of life and functioning?**
Yes / Partially / No

**Is the patient becoming more regulated and adaptable over time?**
Yes / Partially / No

**Are there warning signs of incomplete integration or delayed destabilization?**
Yes / No

**Should the phase of care be maintained or revised?**
Maintain current phase / Step down / Step up / Return to Stabilization or Foundation Building

**Current treatment emphasis:**
Integrate / Maintain / Reassess / Stabilize

**Clinical Principle**

The ethical responsibility of systems-based care extends beyond the intervention itself. Clinicians should monitor not only whether a treatment produced an acute effect, but whether that effect is safely consolidated into the patient’s life.

**V. Ethical and Implementation Safeguards**

**Purpose**

A systems-based multiaxial approach changes the ethical frame of psychiatric treatment. The clinician is not only selecting an evidence-based intervention for a diagnosis; the clinician is judging timing, readiness, intensity, safety, and consolidation across a dynamic human system.

This is especially important for interventions that may temporarily destabilize existing patterns, increase suggestibility, intensify emotion, alter meaning, or loosen rigid psychological organization. Such interventions may be beneficial when the patient has sufficient stability, support, and capacity for integration. They may be harmful when introduced into a system that is unsafe, chaotic, unsupported, or insufficiently prepared.

The central ethical question is therefore not only, “Can this treatment help?” but also: **Can this patient safely tolerate this intervention now, and are the conditions present for adaptive change to consolidate?**

**Informed Consent**

Informed consent should address more than procedural risks or expected benefits. It should explain the phase-of-care rationale for the intervention and clarify why a given treatment is being recommended, deferred, modified, or sequenced after preparatory work.

Informed consent should include discussion of:

- The patient’s current phase of care
- Why the proposed intervention fits that phase
- Potential benefits and limitations
- Acute and delayed risks
- Alternatives, including less intensive options
- The role of preparation and integration
- The importance of follow-up
- The possibility that treatment may need to pause or shift if destabilization occurs
- Boundaries, power dynamics, dependency risk, and suggestibility
- The patient’s right to decline or defer treatment

**Ethical Use of Phase-of-Care Determination**

The phase-of-care designation should guide treatment recommendations, but it should not be used rigidly or punitively. A determination that a patient requires Stabilization or Foundation Building is not a denial of care. It is a clinical judgment that the safest and most effective next step is to increase adaptive capacity before introducing greater intensity.

Similarly, Perturbation-Ready status should not imply that a patient must pursue psychedelic therapy, ketamine-assisted therapy, trauma processing, or any other intensive intervention. It means only that the patient appears to have sufficient stability and support to consider such work if clinically indicated, legally available, and ethically appropriate.

**Core Clinical Competencies**

Clinicians working within a systems-based multiaxial model require competencies that extend beyond diagnosis and protocol delivery. These include:

- Multiaxial formulation across Quality of Life, Body, Mind, and Spirit
- Assessment of Regulation/Adaptability, rigidity, instability, and adaptive capacity
- Phase-of-care determination and revision over time
- Treatment sequencing judgment
- Risk assessment and stabilization planning
- Recognition of decision modifiers
- Assessment of psychological capacities and resources
- Relational containment and alliance repair
- Preparation, integration, and long-horizon monitoring
- Ethical attention to power, dependency, boundaries, and suggestibility
- Capacity to defer intervention when needed
- Collaboration across disciplines

**Team-Based and Stepped-Care Implementation**

Because the four axes often require different forms of expertise, systems-based care is well suited to team-based clinical practice. Psychiatric diagnosis and medication management, psychotherapy, integrative or functional medicine, nutritional care, neurophysiological assessment, mindfulness-based practices, family work, and community support may each address different constraints on adaptive capacity.

A multiaxial model also aligns with stepped care. Not every patient requires intensive care, and not every patient who desires intensive treatment is ready for it. The phase-of-care determination helps clinicians decide when to step up, step down, stabilize, build foundations, introduce perturbation, support integration, or maintain gains.

Implementation may include:

- Screening and measurement using existing validated tools
- Low-intensity stabilization and education
- Skills-based Foundation Building
- Medical or psychiatric stabilization when indicated
- Psychotherapy or relational support
- Perturbation-oriented interventions when appropriate and legal
- Integration and maintenance support
- Reassessment and step-up care when risk increases

**Implementation Safeguards**

Any setting using a systems-based model should establish basic safeguards:

- Clear inclusion and exclusion criteria for intensive interventions
- Phase-of-care documentation
- Defined stabilization pathways
- Emergency and escalation procedures
- Explicit boundary policies
- Informed consent processes
- Integration and follow-up expectations
- Team consultation for complex cases
- Monitoring of delayed adverse effects
- Attention to equity, access, culture, and legal context

**Low-Resource and Global Adaptation**

A systems-based multiaxial framework does not depend on a single proprietary intervention or technology. It can be adapted to specialty clinics, community mental health settings, integrative practices, psychedelic services where legal, primary care collaborations, inpatient or intensive programs, and low-resource environments.

In low-resource settings, the decisive variable is not access to a specific intervention, but the ability to assess readiness, reduce destabilizing constraints, strengthen adaptive capacity, and support consolidation using available resources. Even without advanced technology or specialty interventions, clinicians can use the framework to avoid premature destabilization, prioritize foundational needs, and support durable change.

The Spirit axis also requires cultural humility. Meaning, purpose, connection, and existential coherence should be assessed without privileging any particular religious, spiritual, or philosophical worldview. The clinical task is not to direct belief, but to understand whether the patient’s meaning system supports flexibility, coherence, connection, and adaptive functioning.

**Clinical and Program-Level Outcomes**

At the implementation level, outcomes should track more than symptom reduction. Programs should evaluate whether care improves quality of life, function, safety, regulation, connection, and durability of gains.

Useful program-level outcomes may include:

- Change in quality of life and function
- Symptom change
- Treatment retention and completion
- Need for higher level of care
- Adverse events, including delayed adverse effects
- Integration follow-through
- Functional recovery
- Patient-defined outcomes
- Equity of access and outcomes across populations

**Clinical Principle**

The clinician’s responsibility is not only to provide access to potentially helpful interventions, but to protect timing, pacing, containment, and integration. In systems-based care, restraint can be as therapeutic as action when the patient’s current phase of care requires stabilization or preparation before deeper change.

**Conclusion**

This appendix translates a systems-based view of psychiatric healing into a practical multiaxial clinical framework. The model preserves the value of DSM diagnosis, validated symptom measures, and evidence-based treatments, while organizing them within a broader formulation of quality of life, biological substrate, psychological patterning, meaning and connection, regulation/adaptability, psychological capacities, and decision modifiers.

Its primary clinical output is a phase-of-care determination: what this person needs next and what the system can safely tolerate now. This output links assessment to treatment sequencing, helping clinicians decide when to stabilize, when to build foundations, when to introduce perturbation, when to support active processing, when to integrate, and when to maintain gains.

The goal is not to replace evidence-based psychiatric practice, but to make it more clinically responsive to how change actually unfolds: dynamically, unevenly, relationally, biologically, psychologically, and existentially over time. A systems-based psychiatry is therefore not defined by any single intervention. It is defined by the capacity to assess the whole system, introduce the right level of change at the right time, and support that change until it becomes embodied in a more flexible, coherent, and functional life.
